# The Role of Nrf2 in Cellular Innate Immune Response to Inflammatory Injury

**DOI:** 10.5487/TR.2009.25.4.159

**Published:** 2009-12-30

**Authors:** Jiyoung Kim, Young-Joon Surh

**Affiliations:** Research Institute of Pharmaceutical Sciences, College of Pharmacy, Seoul National University, 599 Kwanak-ro, Kwanak-gu, Seoul, 151-742 Korea

**Keywords:** Nrf2, Innate immunity, Inflammation

## Abstract

Nuclear factor erythroid derived 2-related factor-2 (Nrf2) is a master transcription regulator of antioxidant and cytoprotective proteins that mediate cellular defense against oxidative and inflammatory stresses. Disruption of cellular stress response by Nrf2 deficiency causes enhanced susceptibility to infection and related inflammatory diseases as a consequence of exacerbated immuneediated hypersensitivity and autoimmunity. The cellular defense capacity potentiated by Nrf2 activation appears to balance the population of CD4^+^ and CD8^+^ of lymph node cells for proper innate immune responses. Nrf2 can negatively regulate the activation of pro-inflammatory signaling molecules such as p38 MAPK, NF-KB, and AP-1. Nrf2 subsequently functions to inhibit the production of pro-inflammatory mediators including cytokines, chemokines, cell adhesion molecules, matrix metalloprotein-ases, COX-2 and iNOS. Although not clearly elucidated, the antioxidative function of genes targeted by Nrf2 may cooperatively regulate the innate immune response and also repress the expression of proinflammatory mediators.

## INTRODUCTION

The inflammatory stress which is implicated in the vast variety of pathogenic conditions, such as sepsis, is balanced by an array of counter-regulatory molecules that attempt to restore immunological equilibrium (Cohen, 2002). Such counter-inflammatory response should occur timely and appropriately to resolve the inflammatory injury. The innate immune system is recognized as the critical first line of host defense for sensing and neutralizing pathogenic infection (Thimmulappa *et al.*, 2006), and dysregulation of innate immune response can result in persistent tissue damage and may propagate further infection. Host factors that regulate innate immunity may hence influence inflammatory response and susceptibility to infection. Few host genetic factors that are vital for controlling inflammation are known, and various studies have identified genes encoding proteins, such as Toll-like receptors (TLRs), tumor necrosis factor-α (TNF-α), lipopolysaccharide (LPS)-binding protein, CD14, and bactericidal/permeability-increasing protein (Thimmulappa *et al.*, 2006), responsible for appropriate innate immune response.

Reactive oxygen species (ROS) are important mediators of inflammation. Nuclear factor-erythroid 2 (NF-E2)-related factor 2 (Nrf2) that belongs to the CNC ("cap 'n' collar") subfamily containing the basic leucine zipper region plays a pivotal role in cellular redox balance and stress response (Kobayashi and Yamamoto, 2005). Nrf2 has a pleiotropic role in regulating the constitutive and inducible expression of a battery of antioxidant and other cytoptotective genes by binding to the *cis*-acting enhancer sequence referred to as antioxidant response element (ARE) or electrophile response element (EpRE) (Kensler *et al.*, 2007). The Nrf2-responsive antioxidant enzymes play roles in cellular defense by enhancing the removal of cytotoxic electrophilic species or ROS (Lee and Johnson, 2004).

Besides its role in cellular protection against electrophilic and oxidative stresses, many recent studies have demonstrated that Nrf2 responds to inflammatory stimulations and rescues cells/tissues from inflammatory injuries. Because of its anti-inflammatory as well as antioxidant functions, Nrf2 is known as an important therapeutic target for the treatment and prevention of inflammation-associated disorders (Kim *et al.*, 2009). The importance of Nrf2 as a novel regulator of the innate immune response by protecting against oxidative/inflammatory stress has been recently demonstrated in several animal models. Thus, disruption of this redox-sensitive transcription factor dramatically increases the mortality of mice in response to experimentally-induced septic shock. LPS as well as TNF-$\alpha$ stimulus resulted in greater lung inflammation in Nrf2-deficient mice (Kolls, 2006; Thimmulappa *et al.*, 2006). Likewise, intraperitoneal administration of LPS caused expression of proinflammatory cytokines (e.g., TNF-$\alpha$ and IL-6) and enzymes (COX-2 and iNOS) more in the retina and iris-ciliary body of Nrf2-deficient mice than in the wild-type animals (Nagai *et al.*, 2009). More recently, Reddy and colleagues (2009) have reported impaired innate immunity against bacterial infection following hyperoxia exposure in Nrf2-deficient mice.

As oxidative stress caused by overproduction and/or inefficient elimination of ROS is implicated in diverse human diseases associated with infection and inflammation, understanding the mechanism by which the Nrf2 protects against oxidative damage can provide the therapeutic and preventive strategies. This review mainly focuses on the role of Nrf2-modulated enzymes in innate immunity and inflammation.

## OXIDATIVE STRESS-INDUCED ACTIVATION OF NRF2

In the resting state, the nuclear level of Nrf2 is low as it is sequestered and also degraded in the cytoplasm by the cytoskeleton-associated protein, Kelch-like ECH-associated protein 1 (Keap1) (Fig. 1) (Kensler *et al.*, 2007; Kobayashi and Yamamoto, 2005). Keap1 functions as a negative regulator of Nrf2 by modulating ubiquitination and proteasomal degradation of Nrf2

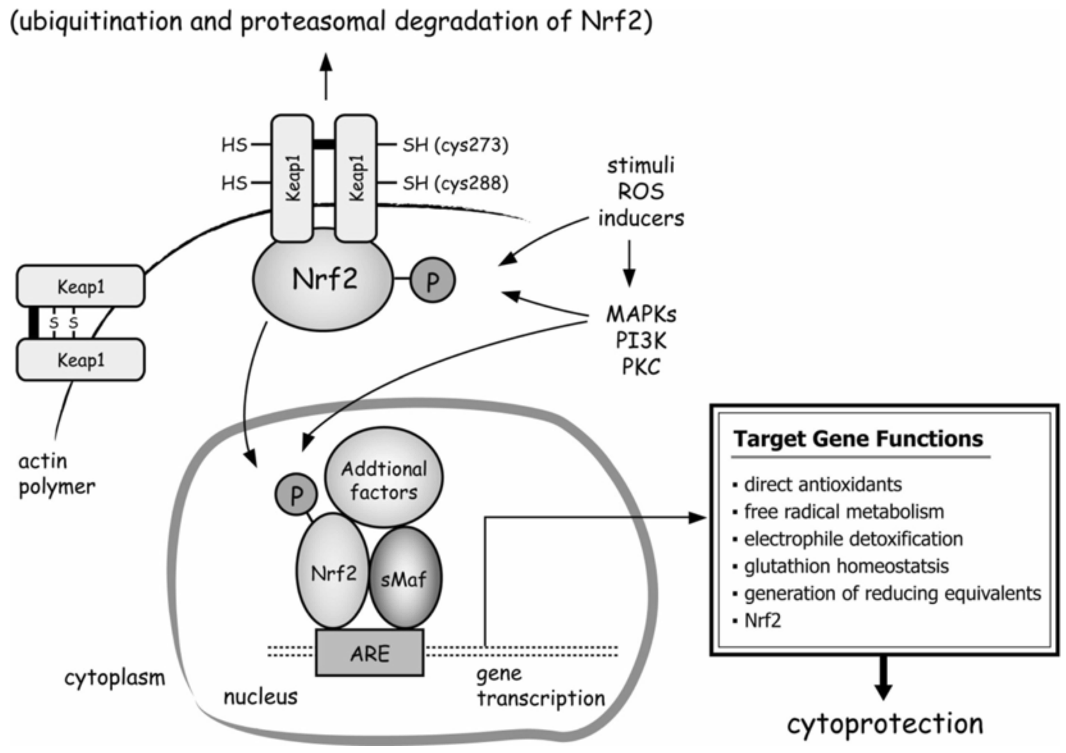

**Fig. 1.** Nrf2-Keap1 signaling. The cytoplasmic repressor Keap1, bound to actin filaments, acts as a sensor for oxidative and electrophilic stresses through cystein residues. In the absence of stimuli, Keap1 sequesters the transcription factor Nrf2 and acts as an adaptor for Nrf2 ubiquitination and proteasomal degradation. Once electrophiles, ROS or ARE inducers intereact with sulfhydryl groups of cysteins in Keap1, ubiquitination switches from Nrf2 to Keap1, leading to the degradation of Keap1 and stabilization and concurrent activation of Nrf2 protein. The phosphorylation of Nrf2 at serine and threonine residues by kinases such as MAPKs, PI3K and PKC is speculated to facilitate the dissociation of Nrf2 from Keap1 and subsequent translocation to the nucleus. After translocation to nucleus, Nrf2 transactivates the expression of genes, which contain AREs in their promoter region. The major function of gene products induced by Nrf2 is cellular detoxification, protection against oxidant damage and regulation of GSH biosynthesis and utilization.

(Kobayashi *et al.*, 2004). Oxidative stress facilitates the dissociation of Nrf2 from Keap1 and subsequent translocation of Nrf2 into the nucleus where it forms a heterodimer with a small Maf (sMaf) protein (Kensler *et al.*, 2007). The Nrf2-sMaf dimer then binds to ARE/EpRE, a *cis*-acting DNA regulatory element with a core nucleotide sequence of 5'-GTGACNNNGCN-3', resulting in enhanced transcriptional activation of target genes, which in turn confers adaptive survival response to ongoing or subsequent stresses (Fig. 1).

A widely accepted model for nuclear accumulation and activation of Nrf2 upon oxidative stress involves alteration of the Keap1 structure by oxidation of cysteines (Cys) contained in Keap1 (Fig. 1). When cells are subjected to abnormally elevated ROS, the reactive Cys residues of Keap1 undergo oxidation and form an intramolecular disulfide bond (Na and Surh, 2006). In this context, reactive sulfhydryl groups present in some critical Cys residues of Keap1 function as sensors for ROS (Dinkova-Kostova *et al.*, 2002). Alternatively, ROS can activate Nrf2 directly by phosphorylating the specific serine and threonine residues of this transcription factor. The following session deals with Nrf2-induced transcriptional activation of some representative genes whose protein products are involved in innate immunity and protection against inflammatory damage.

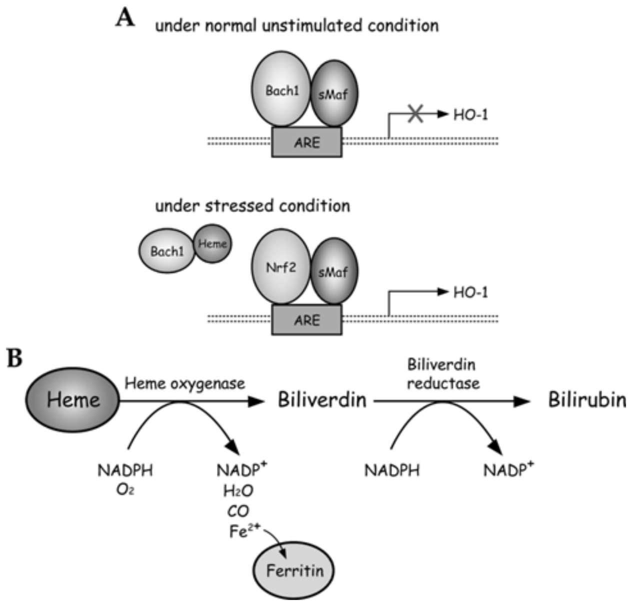

**Fig. 2.** (A) A schematic model for de-repression of Bach1 by heme. Under the normal condition, a heterodimer of Bach1 and sMaf is bound to ARE, and transcription of HO-1 is repressed. Under conditions provoking oxidative stress, heme is released from hemeproteins. The released free heme binds to Bach1 via its heme binding motif and displaces Bach1 from ARE. Once ARE is relieved from Bach1-derived repression, Nrf2 replaces Bach1, forming the heterodimer with sMaf and binding to ARE to stimulate the transcription of HO-1 (Sun *et al.*, 2002; Srisook *et al.*, 2005). (B) Heme catabolism pathway. HO-1 oxidizes heme to form CO, iron ($Fe^{2+}$) and biliverdin. Biliverdin is then converted to bilirubin by biliverdin reductase. Iron ($Fe^{2+}$) quickly binds to ferritin, a ubiquitous iron storage protein, which is also induced by Nrf2.

## NRF2-MEDIATED INDUCTION OF ANTIOXIDANT ENZYMES AND THEIR ROLES IN INNATE IMMUNITY AND ANTI-INFLAMMATION

***Heme oxygenase (HO-1).*** HO-1 has both antioxidant and anti-inflammatory properties. HO-1 promoter contains ARE, and the activation of Nrf2 enhances HO-1 expression in several cell types (Kim *et al.*, 2009). It was reported that regulation of the HO-1 gene expression by Nrf2 is based on the availability of free-heme (Srisook *et al.*, 2005). Under normal cellular conditions, a mammalian transcriptional repressor, Bach1 heteromerizes with sMaf, binds to ARE, and subsequently interferes with Nrf2-binding to ARE. However, under conditions producing oxidative/nitrosative stresses, cellular hemeprotein releases heme, which preferentially binds to Bach1 at its heme-binding motif and thus hampers Bach1 binding to ARE (Srisook *et al.*, 2005). Free heme-derived de-repression of Bach1 was suggested to facilitate Nrf2 to activate the transcription of HO-1 gene (Fig. 2A) (Sun *et al.*, 2002; Srisook *et al.*, 2005).

Protein-bound heme catalyzes electron transfer reactions in cells and is integral to aerobic life. On the other hand, the free heme released as a consequence of degradation of heme proteins by oxidative stress is toxic, because it can catalyze the Fenton reaction and cause oxidative damage to cellular macromolecules. In this respect, HO-1 plays an important role in removal of the cytotoxic pro-oxidant free heme accumulated in cells. HO-1 catalyzes the oxidation of free heme and generates equimolar amounts of ferrous iron ($Fe^{2+}$), carbon monoxide (CO), and biliverdin (Wagener *et al.*, 2003). Biliverdin is then reduced in bilirubin, a lipid-soluble antioxidant, by biliverdin reductase, in mammals (Fig. 2B) (Wagener *et al.*, 2003). Pro-oxidant $Fe^{2+}$ produced by HO-1 is quickly eliminated by iron storage protein ferritin, which is induced by Nrf2 as well (Wagener *et al.*, 2003; Chen and Kunsch, 2004). The heme-derived metabolites, including CO, biliverdin and bilirubin generated by HO-1, have been shown to scavenge ROS such as lipid peroxyl radicals (LOO˙/LOOH), superoxide anion ($O_2^{\cdot-}$), hydrogen peroxide ($H_2O_2$), and peroxynitrite ($ONOO^-$) (Wagener *et al.*, 2003).

Recent research has revealed that HO-1 is a critical modulator of the innate immunity and inflammation. For instance, HO-1 levels are frequently elevated in acute inflammatory disorders, and it was suggested that monocyte HO-1 production confers potent anti-inflammatory effects against excessive cell or tissue injury caused by oxidative stress and cytokinemia (Yachie *et al.*, 2003). Up-regulation of HO-1 ameliorated the inflammatory damages in an endotoxic shock model (Tamion *et al.*, 2007), airway mucus (Almolki *et al.*, 2008), vascular endothelium (Lin *et al.*, 2008), colon (Horvath *et al.*, 2008), and brain (Cuadrado and Rojo, 2008; Syapin, 2008). Moreover, HO-1 attenuated skin inflammation and contact hypersensitivity, and also accelerated wound healing after epithelial injury in mice (Pae *et al.*, 2008; Patil *et al.*, 2008).

The antioxidative properties of heme-derived metabolites CO, biliverdin, and bilirubin were suggested to account for potent anti-inflammatory effects of HO-1. CO was reported to inhibit LPS-induced activation of endothelial cells (Sun *et al.*, 2008a). CO attenuated the inflammatory insult in the liver of thermally injured mice (Sun *et al.*, 2008b). Treatment with a CO-releasing molecule ameilorated joint inflammation and erosion in collagen-induced murine arthritis (Ferrandiz *et al.*, 2008) and enhanced the host defense response to microbial sepsis in mice (Chung *et al.*, 2008). The HO-1/CO-biliverdin pathway was found to down-regulate neutrophil rolling, adhesion and migration in acute inflammation (Freitas *et al.*, 2006). Biliverdin was shown to protect against polymicrobial sepsis (Overhaus *et al.*, 2006), endotoxin-induced acute lung injury (Sarady-Andrews *et al.*, 2005) and colitis (Berberat *et al.*, 2005). Bilirubin, another metabolite derived by HO-1, also exerts an important role in innate immunity and inflammatory response. Bilirubin suppressed experimental autoimmune encephalomyelitis and autoimmune hepatitis (Sawada *et al.*, 1997; Liu *et al.*, 2003). It also inhibited iNOS expression and NO production and protected against endotoxic shock in mice and rats (Wang *et al.*, 2004; Lanone *et al.*, 2005; Kadl *et al.*, 2007). Bilirubin attenuated vascular endothelial activation and dysfunction and inhibited VCAM-1-mediated transendothelial leukocyte migration (Kawamura *et al.*, 2005; Keshavan *et al.*, 2005).

An important cross-talk between HO-1 and the TLR system was reported (Shen *et al.*, 2005; Tsuchihashi *et al.*, 2007). Over-expression of HO-1 down-regulated the activation of STAT-1 via the type-1 interferon (IFN) pathway, downstream of TLR-4, indicating that HO-1 exerts adaptive, cytoprotective and anti-inflammatory functions in the context of innate TLR-4 activation (Tsuchihashi *et al.*, 2007). In studying the mechanism of innate immunity by which HO-1 attenuated the pro-inflammatory responses that are triggered via TLR4 signaling, it was found that TLR4 also negatively modulated HO-1 expression (Shen *et al.*, 2005). In many cases, compounds which enhance the expression of HO-1 also inhibit the pro-inflammatory activation of NF-κB (Chaea *et al.*, 2007; Pang *et al.*, 2008). In fact, HO-1 overexpression, HO-1 induction, or treatment with heme metabolites reversed interleukin (IL)-18-mediated activation of p38 mitogen-activated protein kinase (MAPK) and NF-κB in endothelial cells (Zabalgoitia *et al.*, 2008). HO-1 inhibited the expression of adhesion molecules associated with endothelial cell activation by removing iron and ROS and blocking NF-κB RelA phosphorylation at serine 276 (Seldon *et al.*, 2007). HO-1 activity also influences regulatory T cells in the lungs and prevents smoke-induced B-cell infiltrates (Brandsma *et al.*, 2008). HO-1 augmented production of IL-10 and transforming growth factor (TGF)-β and foxp3$^+$CD4$^+$CD25$^+$ Treg cell function, thereby leading to attenuation of airway inflammation (Xia *et al.*, 2007).

***NAD(P)H:quinone oxidoreductase (NQO1) and NRH:quinone oxidoreductase (NQO2).*** Nrf2 is a major transcription factor regulating the gene expression of NQO1 and NQO2 (Venugopal and Jaiswal, 1996; Wang and Jaiswal, 2006). NQO1 and NQO2 are cytosolic flavoproteins that oxidize NAD(P)H and NRH, respectively, and catalyze two-electron reduction of quinones to less reactive forms of these molecules (hydroquinones) (Fig. 3) (Ross, 2004; Halliwell and Gutteridge, 2007). The obligatory two-electron reduction of quinones catalyzed by NQO1 competes with the one-electron reduction catalyzed by cytochrome P450 reductases and other flavoprotein enzymes, which generate unstable semiquinones (Jaiswal, 2000). NQO1 also keeps coenzyme Q (ubiquinone) in a reduced antioxidant state (ubiquinol) to retain membrane-stabilizing activity (Ross *et al.*, 2000). Ubiquinols can readily inactivate the oxygen free radicals and thus, prevent the oxygen free radical-derived damage to biomolecules and also prevent the initiation of lipid peroxidation (Niki, 1997). The NQO1 and NQO2 proteins hence detoxify endogenous and exogenous quinones and their derivatives, thereby preventing oxidative stress that may arise as a result of their redox cycling (Ross, 2004).

Both NQO1-null and NQO2-null mice showed enhanced susceptibility to autoimmune diseases and predisposition to collagen-induced arthritis (Iskander *et al.*,

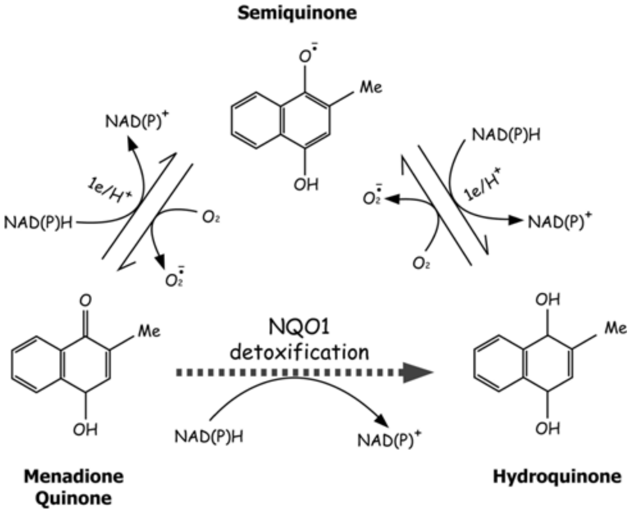

**Fig. 3.** Detoxification of quinone by NQO1. Metabolism of menadione (vitamin K3) by an one-electron (1e/$H^+$) reducing enzyme generates an unstable semiquinone radical, with further one-electron (1e/$H^+$) reduction to the stable hydroquinone. Back oxidation generates ROS ($O_2^-$) when oxygen is present. NQO1 metabolizes menadione by one-step, two-electron (2e/2$H^+$) reduction directly to the hydroquinone, with no ROS production. Inhibition of NQO1 may cause preferential metabolism of menadione by one-electron reductive processes leading to production of unstable semiquinone radical which generates ROS (Criddle *et al.*, 2007).

2006). The double knockout mice deficient in NQO1 and NQO2 showed the increased number and the size of bronchial-associated lymphoid tissue (BALT) with age and exacerbated infiltration of neutrophils and macrophages in BALT (Das *et al.*, 2006). These mice also exhibited significantly increased levels of cytokines, such as TNF-$\alpha$, IL-6, and IL-1$\beta$ in the serum, and iNOS and nitric oxide (NO) in lung macrophages (Das *et al.*, 2006). The disruption in intracellular redox balance resulting from the loss of NQO1 and NQO2 was suggested to cause alterations in the levels of cytokines and chemokines that control inflammation and homing of lymphocytes and neutrophils (Das *et al.*, 2006). Transfecting the airway epithelial cells with NQO1 reduced diesel extract-induced chemokine IL-8 production (Ritz *et al.*, 2007). A close correlation between NQO1 induction and suppression of pro-inflammatory iNOS was observed in murine hepatoma cells, indicating that these processes are functionally linked (Dinkova-Kostova *et al.*, 2005b).

***Glutamate cysteine ligase (GCL).*** Nrf2 plays a key role in modulating the intracellular reduced glutathione (GSH) level by regulating the expression of GCL in aerobic cells (Rahman, 2005). GCL is composed of catalytic (GCLC) and regulatory (GCLM) subunits and the promoter (5'-flanking) regions of both GCLC and GCLM contain ARE (Rahman, 2005). Nrf2-deficient mice exhibited nullified expression of GCL and decreased cellular GSH levels (Chan and Kwong, 2000; Thimmulappa *et al.*, 2006). GSH synthesis in aerobic cells involves two enzymatic steps catalyzed sequentially by GCL and GSH synthetase (Fig. 4A) (Rahman, 2005). The amount and the activity of GCL actually controls the rate of GSH synthesis and the latter enzyme apparently has no regulatory role, since once $\gamma$-glutamyl-cysteine is synthesized, it is rapidly converted to the tripeptide GSH ($\gamma$-glutamyl-cysteinyl-glycine). Although the heavy subunit (GCLC) of GCL retains the entire catalytic activity, its activity can be modulated through association with the regulatory GCLM. It has been calculated that 80% of the cytosolic GCL proteins are inactive under physiological conditions due to its interaction with GSH. Thus, a decrease in GSH triggers less binding of GSH to GCL, resulting in the increased level of physiologically active GCL, and hence enhances the GSH synthesis (Fig. 4A).

GSH as the most abundant, intra- and extra-cellular non-protein thiol buffers changes in the cellular redox status, scavenges ROS, detoxifies electrophilic compounds, and reduces peroxides and protein disulfides (Halliwell and Gutteridge, 2007). GSH also serves as a cofactor in several enzymatic reactions (Halliwell and Gutteridge, 2007). GSH deficiency and resulting disrupted redox-balance are found in many inflammation-mediated diseases, including sepsis (Villa *et al.*, 2002), cystic fibrosis (Tirouvanziam *et al.*, 2006), and rheumatoid arthritis (Hassan *et al.*, 2001). GSH depletion *in vivo* exacerbated pleurisy (Cuzzocrea *et al.*, 1999). GSH rescued mice from lethal sepsis by limiting inflammation and potentiating host defense (Villa *et al.*, 2002). The GSH precursor, *N*-acetyl-cysteine (NAC), prevented ROS damage in the initial phase of septic shock in humans (Ortolani *et al.*, 2002). NAC also augmented the migration of neutrophils in response to infection, decreased bacterial colonies and improved survival in a mouse model of sepsis (Villa *et al.*, 2002). GSH therapies were effective in cystic fibrosis patients by restoring the oxidant-antioxidant balance in the lung epithelial surface (Roum *et al.*, 1999), suppressing sputum IL-8 (Tirouvanziam *et al.*, 2006), lowering the bronchoalveolar lavage fluid (BALF) prostaglandin $E_2$ ($PGE_2$) level (Hartl *et al.*, 2005), and increasing $CD4^+CD8^+$ lymphocytes in BALF with improved lung function (Hartl *et al.*, 2005). An elevated GSH level ameliorated bronchial asthma by altering the T cell type 1 (Th1)/Th2 imbal-

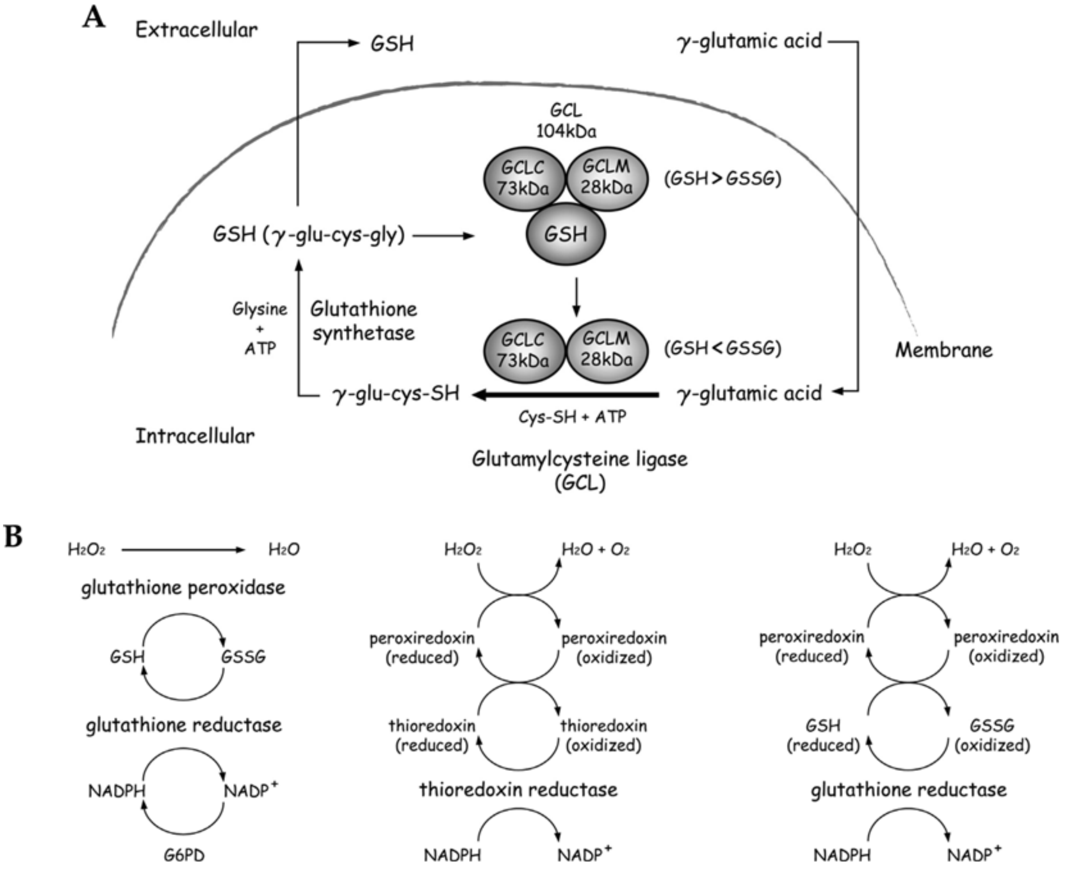

**Fig. 4.** (A) Schematic diagram for GSH synthesis. GSH biosynthesis involves two enzymatic steps catalyzed by GCL and glutathione synthetase (Rahman, 2005). Among the cytosolic GCL proteins, 80% is inactive under physiological conditions due to binding to GSH. A decreased GSH level triggers less binding of GSH to GCL, makes GCL physiologically active, and hence enhances GSH synthesis. GSH synthetase rapidly converts GCL-ligased γ-glutamyl-cysteine to γ-glutamyl-cysteinyl-glycine, GSH. (B) Action of GSH and TRX-dependent enzymes regulated by Nrf2. GSH peroxidase metabolizes $H_2O_2$, generating $H_2O$ and oxidized GSH (GSSG). GSH reductase regenerates GSH. Peroxiredoxin uses GSH and/or TRX as an electron donor for peroxidation of $H_2O_2$, resulting in generation of GSSG and/or oxidized TRX, respectively. Oxidized glutathione (GSSG) and/or oxidized TRX are converted to their reduced forms by GSH reductase and/or TRX reductase, respectively.

ance and suppressing chemokine production and eosinophil migration (Koike *et al.*, 2007). A probiotic able to release GSH prevented colonic inflammation and ameliorated the production of TNF-α and NO in rat colitis (Peran *et al.*, 2006). GSH was also found to ameliorated the damage caused by *H. pylory* infection (Matthews and Butler, 2005).

Since the severity of inflammatory damage is associated with the ability of cells to counteract oxidative stress, modulation of ROS accumulation by GSH may alter the activities of redox-sensitive JNK and p38 MAPK as well as NF-κB and AP-1 and thereby can attenuate pro-inflammatory responses (Rahman and MacNee, 2000). GSH depletion led to enhanced susceptibility to oxidative stress, increased inflammatory cytokine release and impaired T cell-responses (Hartl *et al.*, 2005). Over-expression of GCL in rat hepatoma cells completely inhibited TNF-α-induced activation of NF-κB, AP-1 and JNK (Manna *et al.*, 1999). GSH negatively regulates TNF-α-induced p38 MAPK activation and RANTES production in human bronchial epithelial cells (Hashimoto *et al.*, 2001). Enhanced GSH synthesis inhibited the activation of NF-κB and the release of TNF-α in LPS-stimulated alveolar type II (AT-II) epithelial cells of rat lung (Zhang *et al.*, 2008). GSH-mediated inhibition of p38 activation was also observed in LPS-stimulated rat peritoneal macrophages, together with lowered iNOS and COX-2 expression (Sun *et al.*, 2006).

It was reported that GSH-mediated redox-status determines Th1/Th2 balance in innate immune response (Peterson *et al.*, 1998; Murata *et al.*, 2002a, 2002b). In general, the Th1 pattern is characterized by IL-12 and interferon-γ (IFN-γ) production and the Th2 response pattern is characterized by IL-4, IL-10 and IL-13 production (Romagnani, 1994). GSH depletion inhibited Th1-associated cytokine production and/or favored Th2-associated responses (Peterson *et al.*, 1998). Increases in GSH attenuated Th2-responses including production of IL-4 (Jeannin *et al.*, 1995) and IL-12 (p70) (Koike *et al.*, 2007). IFN-$\gamma$ predominated when the GSH level was high, but declined when GSH was depleted (Romagnani, 1994). In a murine asthma model, intraperitoneal injection of the GSH precursor enhanced the production of IL-12 and IFN-$\gamma$, but reduced the levels of IL-4, IL-5, IL-10 and the chemokines eotaxin and RANTES in BALF (Koike *et al.*, 2007).

***GSH and thioredoxin (TRX)-dependent enzymes.*** GSH and TRX-dependent enzymes (i.e., GSH peroxidase, GSH reductase, GSH *S*-transferase, peroxiredoxin, and TRX reductase) are coordinately regulated by the Nrf2-ARE signaling (Chen and Kunsch, 2004). GSH and TRX systems constitute the major cellular reducing power and free radical scavenging activity in the body. GSH peroxidase and peroxiredoxin metabolize $H_2O_2$, generating $H_2O$ and oxidized glutathione (GSSG) and/or oxidized thioredoxin (Fig. 4B). GSH reductase and TRX reductase regenerate GSH and TRX, respectively, for GSH peroxidase and peroxiredoxin to reuse them. The coordinated regulation of genes encoding aforementioned enzymes by Nrf2 may provide a synergistic effect on maintaining the proper levels of GSH and TRX, eliminating ROS, and subsequently attenuating the inflammation processes.

Lack of GSH peroxidase accelerated inflammatory response in brain (Flentjar *et al.*, 2002), diabetes-associated atherosclerosis (Lewis *et al.*, 2007) and gastrointestinal inflammation (Chu *et al.*, 2004). The reduced activity of GSH peroxidase was associated with the pathogenesis and severity of asthma (Misso *et al.*, 1996). Transgenic mice over-expressing GSH peroxidase were able to modulate host response during endotoxemic conditions (Mirochnitchenko *et al.*, 2000), and showed significant reduction of chemokines and IL-6 in experimentally induced stroke (Ishibashi *et al.*, 2002). A GSH peroxidase mimetic inhibited LPS-induced COX-2 protein expression in RAW264.7 macrophage cells (Nakamura *et al.*, 2002). The treatment of endothelial cells with GSH peroxidase mimetic prevented TNF-$\alpha$ production and neutrophil-induced endothelial alterations (Moutet *et al.*, 1998) and suppressed TNF-$\alpha$-induced VCAM-1 and ICAM-1 gene expression (d'Alessio *et al.*, 1998).

Peroxiredoxin utilizes redox-active cysteines to reduce peroxides, lipid hydroperoxides, and peroxinitrites (Kisucka *et al.*, 2008). It was found that peroxiredoxin I protects against excessive endothelial activation and atherosclerosis (Kisucka *et al.*, 2008). Peroxiredoxin II negatively regulated LPS-induced inflammatory signaling through modulation of ROS synthesis via NADPH oxidase and therefore, prevented excessive host response to microbial products (Yang *et al.*, 2007).

Thioredoxin-1 (TRX-1) reduced cigarette smoke-induced systemic inflammatory responses, lung inflammation and emphysema in mice (Sato *et al.*, 2008). TRX-1 suppressed airway hyper-responsiveness and prevented the development of airway remodeling by inhibiting production of chemokines and Th2 cytokines in an asthma model (Imaoka *et al.*, 2007). Human TRX-1 ameliorated experimental murine colitis in association with reduced macrophage inhibitory factor (MIF) production (Tamaki *et al.*, 2006). Over-expression of TRX-1 prevented the development of chronic pancreatitis via the suppression of oxidative stress and MCP-1-mediated chronic inflammation (Ohashi *et al.*, 2006). TRX also protected against joint destruction in a murine arthritis model (Tsuji *et al.*, 2006).

***CD36.*** Nrf2 has been shown to regulate the expression of CD36 in murine macrophages (Ishii *et al.*, 2004). Thus, CD36 gene expression was not inducible in Nrf2 knockout mice (Iizuka *et al.*, 2005). Multiple ARE-like sequences were found in the promoter region of murine CD36-encoding gene (GeneBank accession No. AF434766) (Ishii *et al.*, 2004). Agents that increase CD36 expression have anti-inflammatory activities by inducing anti-inflammatory cytokine IL-10 (Parsons *et al.*, 2008). CD36 mediates uptake of oxidatively modified low-density lipoproteins (oxLDL), which is known to cause atherosclerosis (Steinberg, 1997; Ross, 1999). CD36 also enhances phagocytosis of apoptotic neutrophils (Greaves *et al.*, 1998; Steinbrecher, 1999; Asada *et al.*, 2004). Since Nrf2 knockout mice showed the significantly smaller number of macrophages that engulfed neutrophils (Iizuka *et al.*, 2005), Nrf2 may play an important role in neutrophil clearance via CD36 induction and thereby protect against neutrophilic inflammation.

***Secretory leukocyte protease inhibitor (SLPI).*** Nrf2 has been shown to induce the expression of the SLPI gene in macrophages (Iizuka *et al.*, 2005; Ishii *et al.*, 2005). SLPI is a cationic serine protease inhibitor with anti-microbial and anti-inflammatory properties present in large quantities in mucosal fluids, including saliva (Angelov *et al.*, 2004). SLPI is a pivotal endogenous factor necessary for optimal tissue repair including intra-oral wound healing and limiting neutrophil elastase-induced pulmonary inflammation (Bingle and Tetley, 1996; Angelov *et al.*, 2004). Adenoviral delivery of SLPI gene attenuated NF-κB-dependent inflammatory signaling in human endothelial cells and macrophages in response to atherogenic stimuli (Henriksen *et al.*, 2004). Mice lacking SLPI are susceptible to LPS-induced endotoxic shock (Nakamura *et al.*, 2003).

## CONCLUDING REMARKS

The new aspect of Nrf2 in innate immunity and inflammation has been recently highlighted. Innate immune response is known to be influenced by cellular redox state (Kolls, 2006). Nrf2-mediated transactivation of antioxidant and other cytoprotective genes play an important role in cellular defence against infections by reducing the production of pro-inflammatory mediators. CO, biliverdin and bilirubin produced as by-products of reactions catalyzed by HO-1 have anti-inflammatory effects. NQO1, another antioxidant enzyme up-regulated by Nrf2, inhibits cytokine-induced VCAM-1 activation. In several inflammatory pathogenic conditions, a protective role of GSH formed by GCL was observed. GSH peroxidase and peroxiredoxin can eliminate hydrogen peroxide, which otherwise activates pro-inflammatory signaling. Nrf2 was also found to induce neutrophil clearance and uptake of oxLDLs through CD36 induction. Nrf2 induces the expression of SLPI which inhibits NF-κB activation and protects against protease-mediated damage. Endogenous molecules such as 15-deoxy-$\Delta^{12,14}$-prostaglandin $J_2$ (15d-$PGJ_2$) and KGF were found to cause Nrf2 activation, which accounts for their anti-inflammatory potential. In general, the coordinated induction of genes targeted by Nrf2 is essential in mounting the innate immune responses upon inflammatory insult.

Intracellular ROS have a fundamental role in pro-inflammatory responses through the activation of redox-sensitive protein kinases and transcription factors such as NF-κB and AP-1. It has been suggested that Nrf2 may inactivate NF-κB, AP-1 and p38 MAPK that mediate proinflammatory signaling (Table 1). Nrf2 was shown to inhibit the expression of proinflammatory cytokines, chemokines, cell adhesion molecules, MMPs, iNOS, and COX-2. Furthermore, Nrf2 deficiency exacerbates inflammatory responses and severe tissue injuries. The activity of Nrf2 influences the pathogenesis of lupus-like autoimmune diseases, rheumatoid arthritis, asthma, emphysema, gastritis, colitis, atherosclerosis and neuroinflammation. Nrf2 also plays an important role in balancing the population of $CD4^+$ and $CD8^+$ of lymph node cells, and Nrf2 deficiency enhances the production of Th2 ($CD4^+$) cytokine and IgE. An association between polymorphism in the Nrf2 promoter and enhanced susceptibility to acute lung injury and some inflammatory ailments was demonstrated (Reddy *et al.*,

**Table 1.** The relationship between Nrf2 and inflammation-regulating factors such as NF-κB, AP-1 and p38 MAPK

| The evidence that the over-expression of Nrf2 or Nrf2 activity inducers negatively regulates the activation of NF-κB in response to stimulation |
|---|
| · Nrf2 knockout cell increased NF-κB activity in response to LPS or TNF-α (Thimmulappa *et al.*, 2006).<br>· Dithiolethione showed inhibition of LPS-induced NF-κB activity (Karuri *et al.*, 2006).<br>· Benzyl isothiocyanates inhibited LPS/IFN-γ-mediated NF-κB activity (Murakami *et al.*, 2003).<br>· Sulforaphane inhibited LPS-induced NF-κB activation (Heiss *et al.*, 2001).<br>· Phenolic antioxidant, 1,4-dihydroquinone inhibited LPS-induced NF-κB activation (Ma and Kinneer, 2002).<br>· 15d-$PGJ_2$ has been shown to inhibit NF-κB activity (Kim and Surh, 2006).<br>· EGCG inhibited NF-κB activity (Gopalakrishnan and Tony Kong, 2008).<br>· Curcumin inhibited NF-κB activity (Gopalakrishnan and Tony Kong, 2008). |
| **The evidence that the over-expression of Nrf2 or Nrf2 activity inducers negatively regulates the activation of AP-1 in response to stimulation** |
| · Transfection with PI3K (the upstream kinase activator of Nrf2) expression plasmid inhibited shear-induced AP-1 (c-Jun) expression (Healy *et al.*, 2005).<br>· Resveratrol inhibited TNF-induced activation of AP-1 activity (Manna *et al.*, 2000).<br>· 15d-$PGJ_2$ inhibited AP-1 activity (Kim and Surh, 2006).<br>· Curcumin inhibited AP-1 activity (Gopalakrishnan and Tony Kong, 2008).<br>· Sulforaphane inhibited UVB-induced AP-1 activation (Zhu *et al.*, 2004).<br>· Dithiolethiones inhibited shear-induced JNK2 and AP-1 (c-Jun) expression (Healy *et al.*, 2005). |
| **The evidence that the over-expression of Nrf2 or Nrf2 activity inducers negatively regulates the activation of p38 MAPK in response to stimulation** |
| · The expression of Nrf2, using Nrf2-containing adenovirus, inhibited the activation of p38 MAPK in response to TNF-α (Chen *et al.*, 2006). |

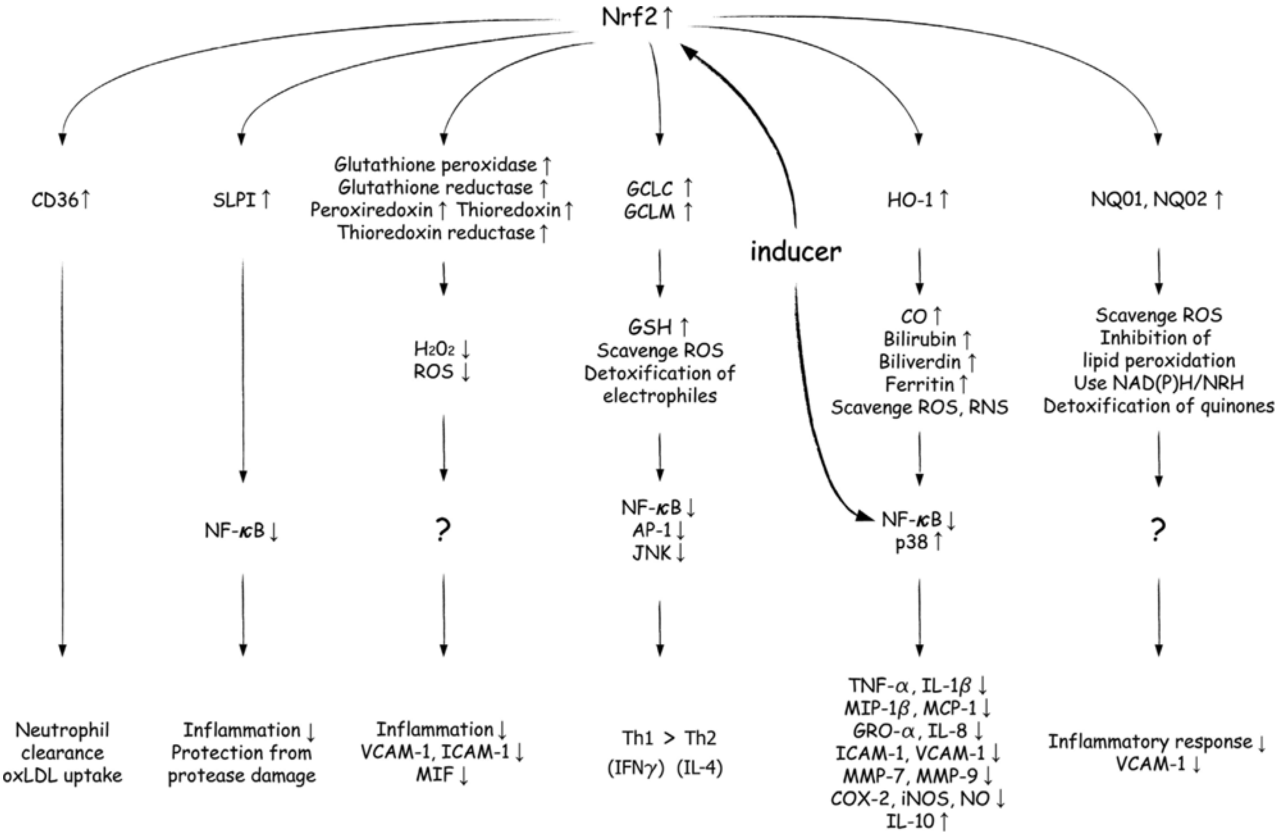

**Fig. 5.** Proposed molecular events of the genes targeted by Nrf2 and their role in innate immunity and anti-inflammation. Innate immunity is the host's first line of defence against infection. However, the defensive innate immune response against infection can be a double-edged sword. The protective process of innate immunity response can develop deleterious excessive inflammation. A delicate balance between innate immunity and inflammation is therefore required, making it possible to fight pathogens effectively while limiting inflammation that might be damaging to the host. Transcription factor Nrf2 might regulate this delicate balance for the proper innate immune response.

2009). Agents activating Nrf2 signaling have been shown to protect against rheumatoid arthritis, gastritis, colitis, atherosclerosis and intracerebral hemorrhage. Adeno-viral gene transfer of Nrf2 was also protective against atherosclerosis and kidney inflammation. Overall, Nrf2 is a master transcription regulator of diverse stress response genes whose protein products protect tissues and cells against infection and inflammation-mediated disease development (Fig. 5). Strong inducers for Nrf2 activation or devices which can efficiently deliver Nrf2 gene to target tissues could be developed as effective therapeutics for inflammation-mediated disorders (Kim *et al.*, 2009).

## ACKNOWLEDGEMENTS

This work was supported by the Innovative Drug Research Center (R11-2007-107-01002-0) from National Research Foundation, Republic of Korea.
